# Long-Term Outcomes of Ahmed Glaucoma Valve Surgery in a Scottish Cohort of Patients With Refractory Glaucoma

**DOI:** 10.7759/cureus.35877

**Published:** 2023-03-07

**Authors:** Thomas Siempis, Osman Younus, Achini Makuloluwa, Donald Montgomery, Catherine Croghan, Sikander Sidiki

**Affiliations:** 1 Ophthalmology, Tennent Institute of Ophthalmology, Gartnavel General Hospital, Glasgow, GBR

**Keywords:** complications, tube shunt surgery, outcomes, ahmed glaucoma valve, refractory glaucoma

## Abstract

Purpose

The purpose of this study was to evaluate the long-term efficacy and safety profile of the Ahmed glaucoma valve (AGV) implantation in cases of refractory glaucoma.

Methods

We conducted a retrospective audit of patients that underwent AGV implantation between 2006 and 2017 by two glaucoma surgeons in a tertiary glaucoma centre (Glasgow, UK). Primary outcome measures included the post-operative intraocular pressure (IOP), number of glaucoma medications, best-corrected visual acuity, complications, re-operation rates, and failure (defined as IOP > 21 mmHg or not reduced by 20% from baseline, IOP ≤ 5 mmHg, reoperation for glaucoma, removal of implant, or loss of light perception) at pre-defined time points (years 1 to 8).

Results

A total of 111 eyes of 94 patients were identified with a mean follow-up of 48.5 months (SD: 31.5); 60.3% of eyes had undergone at least one previous glaucoma surgery. Mean presenting IOP was 31.7 mmHg (SD: 11.4), and it reduced to 13.9 mmHg (SD: 4.2) in year 5 and 16.3 mmHg in year 8 (p<0.05). The number of glaucoma medications reduced from 3.8 (SD: 1.4) to 2.4 (SD: 1.4) and 2.6 (SD: 1.4) in the above time points (p<0.05 except year 8). The five-year success rate was 65.2%, and the five-year reoperation rate was 37% excluding cataract surgery. The most common early complications were hyphaema (12.6%) and transient hypotony (8.1%), whereas the most common late complication was an encapsulated bleb (15.1%).

Conclusions

AGV implantation is an effective procedure for controlling IOP in the long term in cases of refractory glaucoma.

## Introduction

Glaucoma drainage devices (GDDs) offer a key method of controlling intraocular pressure (IOP) in refractory glaucoma [1] Their use has increased in recent years, especially where prior filtration surgery has failed or would be expected to have a low chance of success as a primary procedure such as in uveitic, neovascular, paediatric, and developmental glaucomas [2]. The TVT study provided further evidence that the use of GDDs has been appropriately increasing even in patients with lower risk of surgical failure as its conclusion was that tube shunt surgery had a higher success rate compared to trabeculectomy with MMC during five years of follow-up in patients who had previously undergone trabeculectomy and/or cataract extraction and uncontrolled glaucoma [3].

The Ahmed glaucoma valve (AGV; New World Medical Inc., Rancho Cucamonga, CA, USA) has been in use since 1993 when it received approval from the U.S. Food and Drug Administration [4]. Through the use of a flow restrictor mechanism, designed to maintain IOP of over 8 mmHg, hypotony is less common [4,5]. Its efficacy and complications have been studied and compared to the Baerveldt tube glaucoma implant (Johnson & Johnson Vision, Santa Ana, CA, USA) in two large multi-centre randomised controlled trials: the Ahmed Baerveldt comparison (ABC) study and the Ahmed versus Baerveldt study [6,7]. The cumulative failure rates at five years for the AGV in these studies ranged from 44.7% to 53% [6,7]. Both implants were found to be effective in reducing the IOP, with the Baerveldt tube producing more significant reductions in IOP albeit with higher rates of hypotony when compared to the AGV [6,7]. Most published real-world data looking at the use of AGV come from retrospective observational studies but usually with a relatively short follow-up of three years or less [8-10]. Furthermore, there are limited data comparing the success rates of AGV in eyes with glaucoma that had previously undergone trabeculectomy versus the ones that had previously undergone primary AGV implantation.

The purpose of this study was to describe the long-term outcomes and safety profile of AGV implantation in a Scottish cohort of patients with refractory glaucoma.

## Materials and methods

This was a retrospective observational audit that looked into the records of all patients that underwent AGV implantation between 2006 and 2017 by two glaucoma surgeons in a tertiary glaucoma centre in the city of Glasgow, Scotland, United Kingdom. The indications for AGV implantation were the presence of glaucoma in the context of IOP not controlled medically and/or the presence of glaucoma deemed to be at high risk of surgical failure such as uveitic glaucoma, previous failed filtering surgery, neovascular, aphakic, or congenital glaucoma, and so on. The AGV used was the FP7 model.

Pre-operative examination findings were used as baseline data. Baseline data collection included patients’ demographic data, past ocular history including previous ocular surgery or glaucoma laser treatment, best-corrected distance visual acuity (VA), IOP measurements with Goldmann applanation tonometer, visual field testing, and recording of the number of IOP-lowering medications used. The use of acetazolamide tablets counted as one medication. VA was assessed in the clinic using a Snellen chart and using descriptive terms such as counting fingers, hand motion, light perception, and no light perception for low VA. For our statistical analysis, we converted VA into LogMAR values [11,12].

The primary outcome measures of the study were post-operative IOP and the number of IOP-lowering medications at pre-defined time points (years 1 to 8). Secondary outcomes included any early or late post-operative complications, re-operation rate, and failure rate at five years. We also calculated the survival curves for both the entire cohort and the two subgroups (primary versus secondary AGV implantation).

The criteria for failure were the ones used in the ABC study [6]. These were defined as the presence of IOP > 21 mmHg or less than a 20% reduction below baseline on two consecutive study visits after three months, IOP ≤ 5 mmHg on two consecutive study visits after three months, or any reoperation for glaucoma, loss of light perception, or removal of the implant for any reason. We considered any IOP-related surgical procedure that required taking the patient to the operating theatre as failure. These included cyclodiode laser procedures, flushing of the AGV, excision of Tenon’s capsule around the plate of the AGV, revision of the AGV for the purposes of suboptimal IOP control or hypotony, and the injection of intracameral viscoelastic in cases of hypotony. Bleb needling procedures and/or injection of anti-metabolites performed in the clinic were not counted as failures. For the purposes of survival analysis, eyes that had not failed by the above criteria and were not on IOP-lowering medications were considered complete successes, whereas those that did not fulfil the failure criteria but required medical therapy to stay within the aforementioned IOP levels were defined as qualified successes. Complications within the first month after AGV implantation were defined as early complications. Patients included in the IOP and medication use analyses were censored if they underwent de novo glaucoma procedures such as the ones described above or experienced vision loss to NPL (no perception of light) to prevent confounding.

This type of study does not require ethical committee approval as it is viewed as an audit (http://www.hra-decisiontools.org.uk/research/). All statistical analyses were performed using SPSS statistics package (IBM Corp., Armonk, NY, USA). Univariate comparisons between treatment groups were performed using the two-sided Student t-test for continuous variables. Risk factors for treatment failure were assessed for statistical significance using the Kaplan-Meier survival analysis log-rank test. A p-value of less than 0.05 was considered statistically significant in our analyses.

## Results

Baseline data

A total of 111 eyes of 95 patients were identified. The mean follow-up for the entire group of patients was 48.5 ± 31.5 months (range: 2-96 months). The mean age for all patients was 50.4 years (SD: ±19.1, range: 12-84 years); 53 patients were male (55.8%) and 41 female (44.2%). The most common diagnoses were uveitic glaucoma in 34 (30.6%) eyes, primary open-angle glaucoma (POAG) in 24 (21.6%) eyes, aphakic glaucoma in 13 (11.7%) eyes, congenital glaucoma in 11 (9.9%) eyes, and neovascular glaucoma in 8 (7.2%) eyes. The full patient demographic data are summarized in Table 1.

**Table 1:** Demographics and ocular history data AGV, Ahmed glaucoma valve; GDD, glaucoma drainage device; VF, visual field; POAG, primary open-angle glaucoma

	n	Percentage
Patients	94	
Male	53	55.80%
Female	41	44.20%
Eyes	111	
Mean age at AGV implantation ± SD (range)	50.4 ± 19.1 (12-84)	
Mean deviation of VFs (dB)	-16.9 ± 9.4	
Mean pattern standard deviation of VFs (dB)	7.3 ± 3.4	
Glaucoma aetiology		
Uveitic	34	30.63%
POAG	24	21.62%
Aphakic	13	11.71%
Congenital	9	8.10%
Neovascular glaucoma	8	7.20%
Primary angle closure	5	4.50%
Normal tension glaucoma	4	3.60%
Traumatic glaucoma	3	2.70%
Post corneal transplantation	2	1.80%
Pigmentary glaucoma	2	1.80%
Pseudoexfoliation glaucoma	2	1.80%
Iridocorneal endothelial syndrome	2	1.80%
Mixed mechanism glaucoma	1	0.90%
Secondary angle closure glaucoma	1	0.90%
Steroid-induced glaucoma	1	0.90%
Lens status at the time of surgery		
Phakic	57	51.40%
Pseudophakic	34	30.60%
Aphakic	20	18%
Glaucoma surgeries pre-AGV implantation		
One previous trabeculectomy	45	40.50%
Multiple trabeculectomies	8	7.20%
Previous GDD	5	4.50%
Cyclodestructive procedure	8	7.20%

Mean IOP and number of medications

In Table 2, we present the mean IOP at baseline and at subsequent time points along with the mean number of medications for each time point (years 1 to 8).

**Table 2:** Mean IOP and number of medications at various time points IOP, intraocular pressure

	Mean ± standard deviation	P-value (compared to baseline)
Baseline IOP (mmHg) (n=111)	31.7± 11.4	
Year 1 IOP (mmHg) (n=75)	16±7	0.001
Glaucoma medications	2 ± 1.4	0.001
Year 2 IOP (mmHg) (n=61)	13.9 ±4.2	0.001
Glaucoma medications	2.1 ± 1.3	0.001
Year 3 IOP (mmHg) (n=48)	13.6 ± 4.7	0.001
Glaucoma medications	2.2 ±1.3	0.001
Year 4 IOP (mmHg) (n=43)	14.3 ±4.5	0.001
Glaucoma medications	2.2 ± 1.3	0.001
Year 5 IOP (mmHg) (n=30)	13.9 ±4.2	0.001
Glaucoma medications	2.4 ± 1.4	0.001
Year 6 IOP (mmHg) (n=23)	13.8 ± 3.2	0.001
Glaucoma medications	2.4 ± 1.5	0.008
Year 7 IOP (mmHg) (n=16)	13.9 ± 4.9	0.001
Glaucoma medications	2.1 ± 1.7	0.008
Year 8 IOP (mmHg) (n=7)	16.3± 5.3	0.001
Glaucoma medications	2.6 ± 1.4	0.172

Visual acuity

The mean VA at baseline in the group with at least five years of follow-up (n=44 with VA data) was 0.49 LogMAR (SD: 0.58; median: 0.3; IQR: 0.2-0.5) (versus a mean of 0.55 LogMAR ± 0.34 for the entire cohort). VA at five years was 0.94 LogMAR (SD: 0.96; median: 0.55; IQR: 0.2-1.45). The deterioration in VA was statistically significant at five years (p=0.00021). A total of 24 eyes out of 44 eyes with 5 year data (54.5%) experienced a reduction in VA of 2 lines. Three of these eyes progressed to NPL due to progression of their glaucoma (6.8%).

Reasons for reduction in vision of more than two lines at five years include glaucoma progression in 11 (25%) eyes, cataract in 3 (6.8%) eyes, retinal ischaemia in two (4.5%) eyes due to advanced diabetic eye disease, corneal decompensation in two (4.5%) eyes, recurrent cystoid macular oedema in one (2.2%) eye, posterior capsular opacification in one (2.2%) eye, band keratopathy in one (2.2%) eye, and traumatic globe rupture in one (2.2%) eye. It was not possible to establish the cause of reduced vision from the medical records in two (4.5%) eyes.

Failure rates and survival curves

As far as the failure rate in the entire cohort of 111 eyes is concerned, 37 eyes were considered as failures irrespective of their follow-up, and 34 of these eyes failed within the first five years of follow-up. The reasons for treatment failure (n=37 out of 111 eyes) are presented below.

A total of 24 eyes had at least one re-operation to lower IOP. The most common surgical procedure that counted as failure (in the entire cohort) was valve flushing in 16 eyes and/or cyclodiode laser in 11 eyes. Eight eyes had inadequate IOP control but no additional glaucoma surgery was required. Three eyes had persistent hypotony that required further surgery. One eye had an operation to remove the AGV due to encapsulation and associated rise in IOP. This patient went on to have a trabeculectomy. Two more eyes had an AGV removal but they had already failed prior to this and have been counted above. The first one was due to recurrent encapsulation around the plate of the AGV and this eye too had subsequent augmented trabeculectomy after multiple needling and valve flushings. In the other case, the valve was removed due to recurrent scleritis and associated pain. One eye had loss of light perception.

Full details of all types of procedures performed after the AGV implantation are presented in Table 3.

**Table 3:** Subsequent ocular procedures following AGV implantation AGV, Ahmed glaucoma valve

Procedure	N	Percentage (as a proportion to the entire cohort)	Mean time ± SD (months)
Cataract extraction	17	15.3%	32.4± 30.6
Valve flushing	16	14.4%	32.2 ± 23.9
Bleb needling	11	9.9%	10.8 ± 17.6
Cyclodestructive procedure	11	9.9%	19.4 ± 18.7
Capsular excision	6	5.4%	19.2 ± 26.2
Additional AGV insertion	4	3.6%	34.7 ± 16.7
Valve revision for hypotony	3	6.3%	10.3 ±9.9
AGV removal	3	2.7%	46.8 ± 34.0

The five-year re-operation rate for eyes that had completed at least five years of follow-up (n=46) was 37% (17 out of 46 eyes) excluding cataract surgery and clinic-based procedures such as selective laser trabeculoplasty or 39.1% including cataract surgery.

**Figure 1:**
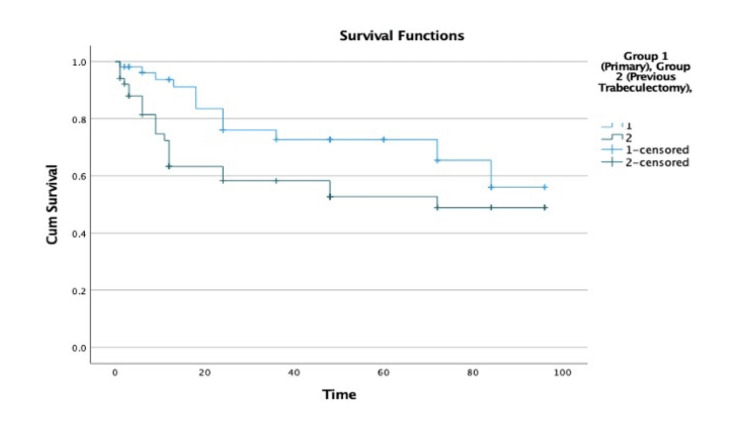
Kaplan-Meier survival curve for overall success stratified for previous ocular surgery: group 1 (primary AGV) and group 2 (previous trabeculectomy). AGV, Ahmed glaucoma valve

In terms of the probability of failure in the above subgroup, 16 of these 46 eyes failed within 60 months in keeping with a failure rate of 34.8% and an overall success rate of 65.2%. Complete success (no requirement for IOP-lowering medical therapy) was noted in five (10.8%) patients at five years. Excluding five eyes which had undergone previous AGV implantation, the mean survival for primary AGV (n=54) was 72 months (SE: 5.54; 95% CI: 61.08 to 82.83 months) and 56.1 months for eyes with previous trabeculectomy surgery (secondary AGV; n=52) (SE: 6.3; 95% CI: 55.8 to 72.5 months), with p = 0.048 (log rank - Mantel Cox). The Kaplan-Meier curves for the primary AGV and secondary AGV groups are presented in Figure 1.

When we compared these two groups (primary versus secondary AGV) in eyes with at least five years of follow-up and excluding those with previous AGV implantation (n=43), four out of 17 eyes with primary AGV failed at five years (23.5%) and 11 out of 26 eyes with secondary AGV (42.3%) failed within five years (p=0.32; Fisher exact test).

Complications

The most frequent early complications (within 1 month) are presented in Table 4.

**Table 4:** Frequency of early post-operative complications

Type of complication	n	Percentage
Hyphaema	14	12.6%
Hypotony	9	8.1%
Choroidal effusions	7	6.3%
Vitreous haemmorhage	4	3.6%
Cystoid macular oedema	2	1.8%
Partial occlusion of the tube with iris	2	1.8%
Diplopia	1	0.9%

The most common late post-operative complications were encapsulated cystic bleb formation in 17 patients and corneal decompensation in six patients (two required corneal transplant surgery). Two cases of persistent hypotony required further surgery to address the issue. There was also one case which required revision for tube exposure. Finally, one patient with a baseline VA of 2.3 LogMAR and background of secondary aphakic glaucoma and chronic uveitis following penetrating injury underwent enucleation of the operated eye nine months following the AGV implantation due to chronic pain in that eye.

## Discussion

The primary purpose of this study was to evaluate the long-term clinical outcomes as well as complication and re-operation rates of the AGV implantation in a heterogenous, in terms of glaucoma-related diagnoses, cohort of patients in a tertiary centre in Scotland. The majority of eyes included in this study had a diagnosis of uveitic glaucoma (30.6%) followed by POAG (21.6%) and aphakic or congenital glaucoma (19.8%), with rubeotic glaucoma seen in 7.2% of the cases. We also explored whether there was any significant difference in the survival curves between primary and secondary AGV implantation. There are not many peer-reviewed published studies with long-term follow-ups on the subject, and we aim to add more data to the literature through this paper.

Our results show that AGV implantation produced both statistically and clinically significant reductions in IOP from a baseline of 31.7 mmHg to a final average of 16.3 mmHg in year 8. It also resulted in a statistically significant and clinically meaningful reduction in the number of IOP-lowering medications used from a baseline of 3.8 to 2.1 in year 7 (p=0.008). These results are similar to the AGV arm of the 2014 ABC study and to the AGV arm of the Ahmed versus Baerveldt Study albeit with higher number of IOP-lowering medications in our cohort at five years (2.4 in our cohort vs 1.8 in the Ahmed versus Baerveldt Study) [6,7]. When comparing our results to other large retrospective studies of AGVs with similarly long follow-up, Luzu et al. reported a baseline mean pre-operative IOP of 31 mmHg that reduced to 17.2 mmHg after a mean follow-up of 35.2 months, whereas Souza et al. reported in their cohort of AGV patients a pre-operative IOP of 30.4 mmHg that reduced to 15.9 mmHg at five years [13,14]. The reported drop in the number of IOP-lowering medications used on these studies was 5.3 reducing at baseline to 2.8 at the conclusion of follow-up in the study by Luzu et al. and 3.2 pre-operatively reducing to 2.1 in month 60 in the study by Souza et al. [13,14]. Additionally, a large Korean study evaluating AGV implantations published in 2017 reported similar reductions in these parameters [15].

One of the caveats when looking at the success rates of any glaucoma operation are the different definitions used in every study. Rotchford et al. examined 100 publications including the term “trabeculectomy” over the period of five years and identified 92 distinct IOP-related definitions of success which when applied to a series of trabeculectomy patients led to a success rate of 36% to 98% over three years of follow-up [16]. At the same time, the IOP targets are dynamic values that tend to be individualised by most glaucoma specialists during follow-up of patients based on their glaucoma progression. Furthermore, the populations studied in various studies tend to be different, and it is difficult to make direct comparisons between studies. The criteria we used for defining what constituted a success and failure were the ones used in the ABC study and are based on consensus definitions contained in the World Glaucoma Association Guidelines on Design and Reporting of Surgical Trials [6,17]. Using these criteria, we reported an overall success rate of 65.2% at five years. Most studies published in the last decade report success rates for the AGV between 45% and 56% at five years. It is worth noting that differences in the studies’ sample size, types, and different proportions of glaucoma-related diagnoses in each cohort as well as different criteria used for success can have an effect on the reported success rate as explained above. More specifically, Lee et al. reported a slightly lower success rate to our results (56% at five years) but in a larger sample of 102 eyes [15]. The majority of eyes in that study had a diagnosis of neovascular glaucoma (39.1%) followed by POAG (16.6%) and uveitis (14.2%) [15]. Additionally, a post-operative IOP between 21 mmHg and 5 mmHg and a 30% rather than 20% reduction from baseline together with no loss of light perception after surgery and no additional filtering surgery or aqueous drainage surgery were considered as a success in that study [15]. Luzu et al. reported lower success rate of 45.1% at five years but similarly used stricter criteria to define failures [13]. The Ahmed versus Baerveldt study reported a success rate of 47% at five years in the AGV group, whereas the ABC study reported findings a five-year success rate of 55.3% [6,7].

The effect of previous ocular surgery on AGV outcomes has not been established. The Tube Versus Trabeculectomy study compared the use of Baerveldt valves to trabeculectomy in eyes with prior ocular surgery [3]. Treatment failure occurred in one-third of patients in the tube group at 5 years. An identical failure rate was reported for the Baerveldt valve, when employed as a primary incisional procedure in the Primary Tube Versus Trabeculectomy study [18]. Both of these studies were well-designed, randomized controlled trials, yet excluded cases of refractory glaucoma. In a cohort of 302 refractory glaucoma patients reported by Lee et al., surgical success rates were not significantly different between eyes with primary (no previous glacuoma surgery) AGV and secondary (previous glaucoma surgery) AGV. [15] Conversely, Souza et al., in a retrospective review of 78 eyes with refractory glaucoma, estimated that prior glaucoma surgery carried a three-fold increased risk of AGV failure [14]. Their cohort included 32% of patients with previous glaucoma surgery. In our study, 47.8% of eyes had a prior trabeculectomy. Our primary AGV failure rate was 23.5%, rising to 42.3% for a secondary AGV implant. We approximated a two-fold increased risk of AGV failure associated with previous trabeculectomy. The Kaplan-Meier survival analysis favoured a survival advantage for primary AGV (Figure 1). This was not statistically significant (p=0.32); however, our study was not powered to test for a hypothetical difference in the success rates between primary and secondary AGV implantation. It is important to note that our study population, comprising inflammatory and neovascular glaucoma, had a high baseline risk of surgical failure. Our results, and previously published studies, cannot be extrapolated to indicate a difference between primary trabeculectomy and AGV implantation in refractory glaucoma. It remains each surgeon's preference to opt for AGV as a primary procedure, whereby it is perceived an eye is at higher risk of trabeculectomy failure [19,20].

The most common complication in our series was an encapsulated bleb formation (15.4%) followed by transient hyphaema (12.6%) and early or late hypotony (9.9%). Late corneal decompensation developed in six (5.4%) patients. Complication rates vary widely between studies. Suza et al. reported incidence of 29% for hypotony, whereas mild-to-moderate hyphema was seen in nine (12%) eyes [14]. Lee et al. have reported much lower incidences of post-operative complications [15]. In the Ahmed versus Baerveldt study, the most common complications post-AGV implantation were shallow anterior chamber (15%), choroidal effusions (13%), tube complications (13%), and clinical bleb encapsulation (11%) [7]. In the ABC study, the most common complications were diplopia (12.7%) and persistent corneal oedema likely attributable to the implant in 11.9% of cases [21]. With respect to the five-year re-operation rate in our case series, this was 37% excluding cataract surgery or 39.1% including cataract surgery. This compares 51% in the Ahmed group in the Ahmed versus Baerveldt study [7].

The changes in VA reported following AGV implantation in refractory glaucoma are variable. We observed that approximately half (54.5%) of AGV eyes lost more than two lines in VA by year 5. Souza et al. reported 13% reduction by two lines or more over the same time period [14]. However, our median reduction in VA was 0.25 logMAR units, consistent with previously published studies reporting reductions of 0.3-0.35 LogMAR units [6,7]. Similarly, our rate of significant visual loss compares favourably to the AGV arm within the Ahmed versus Baerveldt and ABC studies [6,7]. Comparison of VA across studies should be interpreted with caution because of differences in pre-operative VA, and measurement of VA is not consistent.

The current study is limited by its retrospective non-randomised design and smaller numbers in later follow-up time points. Its strengths are that our cohort included complicated cases with multiple risk factors for filtration surgery failure, such as younger age, being aphakic or pseudophakic, and having had multiple previous ocular surgeries including previous failed glaucoma surgery. It is also one of the largest UK-based case series of AGV implantation in a variety of glaucoma diagnoses with a long follow-up and detailed data on complications and re-operation rates.

## Conclusions

In conclusion, our study shows that AGV is a very effective procedure in treating refractory glaucoma, reducing the IOP on average by almost 50% for at least five years post-surgery, while at the same time reducing the number of IOP-lowering medications used. It appears that primary AGVs do better than secondary AGVs, although the results are not statistically significant. The complication and re-operation rates are comparable to other types of filtering surgery. Our results are in keeping with published real-world data from other centres.
